# Fatty liver index is an independent risk factor for all-cause mortality and major cardiovascular events in type 1 diabetes: an 11-year observational study

**DOI:** 10.1186/s12933-024-02171-9

**Published:** 2024-02-28

**Authors:** Monia Garofolo, Daniela Lucchesi, Massimo Giambalvo, Michele Aragona, Alessandra Bertolotto, Fabrizio Campi, Cristina Bianchi, Paolo Francesconi, Piero Marchetti, Stefano Del Prato, Giuseppe Penno

**Affiliations:** 1 Section of Diabetes and Metabolic Diseases, Department of Clinical and Experimental Medicine, University of Pisa, Via A. Trivella, 1, 56126 Pisa, Italy; 2 Department of Medical Specialties, University Hospital of Pisa, Pisa, Italy; 3 Epidemiology Unit, Regional Health Agency (ARS) of Tuscany, Florence, Italy; 4 Sant’Anna School of Advanced Studies, Pisa, Italy

**Keywords:** Type 1 diabetes, NAFLD, Fatty liver index, Mortality, Cardiovascular outcomes

## Abstract

**Background:**

Non-alcoholic fatty liver disease (NAFLD), identified by the Fatty Liver Index (FLI), is associated with increased mortality and cardiovascular (CV) outcomes. Whether this also applies to type 1 diabetes (T1D) has not been yet reported.

**Methods:**

We prospectively observed 774 subjects with type 1 diabetes (males 52%, 30.3 ± 11.1 years old, diabetes duration (DD) 18.5 ± 11.6 years, HbA1c 7.8 ± 1.2%) to assess the associations between FLI (based on BMI, waist circumference, gamma-glutamyl transferase and triglycerides) and all-cause death and first CV events.

**Results:**

Over a median 11-year follow-up, 57 subjects died (7.4%) and 49 CV events (6.7%) occurred among 736 individuals with retrievable incidence data. At baseline, FLI was < 30 in 515 subjects (66.5%), 30–59 in 169 (21.8%), and ≥ 60 in 90 (11.6%). Mortality increased steeply with FLI: 3.9, 10.1, 22.2% (p < 0.0001). In unadjusted Cox analysis, compared to FLI < 30, risk of death increased in FLI 30–59 (HR 2.85, 95% CI 1.49–5.45, p = 0.002) and FLI ≥ 60 (6.07, 3.27–11.29, p < 0.0001). Adjusting for Steno Type 1 Risk Engine (ST1-RE; based on age, sex, DD, systolic BP, LDL cholesterol, HbA1c, albuminuria, eGFR, smoking and exercise), HR was 1.52 (0.78–2.97) for FLI 30–59 and 3.04 (1.59–5.82, p = 0.001) for FLI ≥ 60. Inclusion of prior CV events slightly modified HRs. FLI impact was confirmed upon adjustment for EURODIAB Risk Engine (EURO-RE; based on age, HbA1c, waist-to-hip ratio, albuminuria and HDL cholesterol): FLI 30–59: HR 1.24, 0.62–2.48; FLI ≥ 60: 2.54, 1.30–4.95, p = 0.007), even after inclusion of prior CVD. CV events incidence increased with FLI: 3.5, 10.5, 17.2% (p < 0.0001). In unadjusted Cox, HR was 3.24 (1.65–6.34, p = 0.001) for FLI 30–59 and 5.41 (2.70–10.83, p < 0.0001) for FLI ≥ 60. After adjustment for ST1-RE or EURO-RE, FLI ≥ 60 remained statistically associated with risk of incident CV events, with trivial modification with prior CVD inclusion.

**Conclusions:**

This observational prospective study shows that FLI is associated with higher all-cause mortality and increased risk of incident CV events in type 1 diabetes.

**Supplementary Information:**

The online version contains supplementary material available at 10.1186/s12933-024-02171-9.

## Background

Non-alcoholic fatty liver disease (NAFLD) affects approximatively 25% of the general population reflecting the prevalence growth of obesity, type 2 diabetes, and metabolic syndrome [1–3]. NAFLD encompasses a continuum that ranges from steatosis, with or without mild inflammation (non-alcoholic fatty liver, NAFL), to non-alcoholic steatohepatitis (NASH) characterized by fibrosis progression, which can ultimately lead to cirrhosis, liver failure, and hepatocellular carcinoma [2, 3].

In people with type 2 diabetes the prevalence of NAFLD is at least twofold higher than in the general population [4], ranging from 55 to 70% [2]. Furthermore, subjects with type 2 diabetes are more likely to develop NASH (30–40%) and to progress toward fibrosis and cirrhosis [2, 4]. NAFLD is also associated with increased risk of fatal or non-fatal cardiovascular (CV) events, irrespective of concomitant risk factors, including diabetes [5, 6].

Liver biopsy and/or magnetic resonance imaging (MRI) are considered gold-standard for NAFLD diagnosis, yet ultrasonography is commonly used as first-line diagnostic tool. For population studies, non-invasive scores based on serum biomarkers have been proposed [6, 7]. The Fatty Liver Index (FLI) is a validated, non-invasive score that reliably predicts the presence but not the severity of steatosis. FLI has been shown to correlate with the degree of insulin resistance and to predict metabolic, hepatic, and cardiovascular morbidity and mortality [6, 7].

Less clear is whether NAFLD in people with type 1 diabetes also increase CV risk. This is a relevant question because an increasing proportion of them is overweight and present features of insulin resistance. According to a recent meta-analysis, NAFLD occurs in about 19% of subjects with type 1 diabetes, although this figure is highly dependent on the diagnostic modality ranging from 0% to 64.7% [8]. This analysis did not include FLI as a diagnostic tool, yet in a small study, FLI showed a good performance as a surrogate marker of liver fat [9].

We have then assessed to which extent FLI was associated with CV outcomes and mortality in a single-center type 1 diabetes cohort over a mean 11-year follow-up.

## Methods

### Study design and participants

This is an observational single-center study with prospective assessment of all-cause mortality and CV events in a cohort of individuals with type 1 diabetes. The research design and the study population have been previously described [10]. All subjects with type 1 diabetes (n = 843) attending the Diabetes Outpatient Clinic of the Azienda Ospedaliero-Universitaria Pisana from January 1, 2001 through December 31, 2009 because of their usual screening for diabetic complications were considered eligible. Type 1 diabetes was diagnosed based on age at onset < 36 years and immediate requirement of insulin therapy with unbroken need for insulin after the first year since diagnosis [11]. Pregnant women, participants of not-white ethnicity, those with type 1 diabetes for less than one year, those on dialysis or with renal transplantation, those with a prior history of viral hepatitis or cirrhosis of any etiology as well those with significant alcohol intake (≥ 2 alcohol units per day in men and ≥ 1 alcohol unit per day in women), those with active cancer, and three individuals for whom valid information on vital status could not be retrieved were excluded. Thus, a total of 774 individuals were recruited into the study. The Ethics Committee of the University of Pisa approved the study protocol, consent procedures, and data analysis plan.

Information about onset and duration of diabetes (DD), smoking habits, physical activity, current insulin treatment, concomitant blood pressure- and lipid-lowering therapies were collected at baseline together with the assessment of the presence and severity of micro- and CV complications, as previously reported [10]. Body weight, height, and waist circumference (WC) were obtained for body mass index (BMI) calculation. Blood pressure (BP), taken after 5-min rest in a sitting position, was calculated as the average of at least two consecutive measurements obtained about 5-min apart. Hypertension was defined as systolic BP > 140 mmHg and/or diastolic BP > 80 mmHg and/or the use of any antihypertensive drug.

In all subjects, urine samples were obtained, and blood samples were drawn at study entry after an overnight fast for determination of serum creatinine, HbA1c, total- and HDL-cholesterol, triglycerides, alanine aminotransferase (ALT), aspartate aminotransferase (AST), gamma-glutamyl transferase (GGT), uric acid, fibrinogen, and urinary albumin to creatinine ratio (ACR). Finally, all participants underwent a screening for diabetic complications as previously described [10]. Estimated glucose-disposal rate (eGDR), a proxy of insulin resistance, was calculated based on WC, presence of hypertension and HbA1c, as previously described [12].

### Calculation of FLI and CV risk scores

The Fatty Liver Index (FLI) was calculated as proposed by Bedogni et al. [13] based on triglycerides, GGT, BMI, and WC:

$${\text{FLI}}\, = \,\left( {{{\text{e}}^{0.{\text{953}}*{\text{loge }}\left( {{\text{triglycerides}}} \right){\text{ }} + {\text{ }}0.{\text{139}}*{\text{BMI }} + {\text{ }}0.{\text{718}}*{\text{loge }}\left( {{\text{GGT}}} \right){\text{ }} + {\text{ }}0.0{\text{53}}*{\text{WC}} - {\text{15}}.{\text{745}}}}} \right)/\left( {{\text{1}}\, + \,{{\text{e}}^{0.{\text{953}}*{\text{loge }}\left( {{\text{triglycerides}}} \right){\text{ }} + {\text{ }}0.{\text{139}}*{\text{BMI }} + {\text{ }}0.{\text{718}}*{\text{loge }}\left( {{\text{GGT}}} \right){\text{ }} + {\text{ }}0.0{\text{53}}*{\text{WC}}{-}{\text{15}}.{\text{745}}}}} \right){\text{ }}*{\text{ 1}}00.$$

FLI was categorized as follows: i. FLI < 30: no fatty liver, ii. FLI 30–59: intermediate status, and iii. FLI ≥ 60: hepatic steatosis [13].

To assess to which extent FLI could be associated to overall risk independent of other risk factors we have calculated the Steno Type 1 Risk Engine (ST1-RE) [14]. The ST1-RE is a risk model for composite CV outcome (risk of first fatal or nonfatal CV event: coronary heart disease—CHD—ischemic stroke, heart failure, and peripheral artery disease) in subjects with type 1 diabetes. It is based on age, sex, DD, systolic BP, LDL-cholesterol, HbA1c, albuminuria, glomerular filtration rate, smoking, and exercise [14].

To the same purpose, we also calculated the EURODIAB Prospective Complication Study Risk Engine (EURO-RE) score [15]. The score, based on age, HbA1c, waist-to-hip ratio, ACR and HDL-cholesterol levels, is used to calculate the risk of major CHD, stroke, end-stage renal failure, amputations, blindness, and all-cause death in type 1 diabetes. The performance of both models has been previously validated in independent prospective cohorts.

For both scores, three groups have been defined: low CV score (10-year risk < 10%), intermediate score (10-year risk 10–19%), and high score (10-year risk ≥ 20%).

### Assessment of outcomes

For each patient we searched and recorded major CV events up to December 31, 2017, and all-cause death up to October 31, 2018 [10, 12]. Vital status was available for all participants and was verified by interrogating the Italian Health Card Database (http://sistemats1.sanita.finanze.it/wps/portal/). Data on the incidence of all CV outcomes and coronary events were available for 736 participants (95.1% of the whole cohort) and were obtained, upon data anonymization, in collaboration with the Regional Health Agency of the Tuscany Region through hospital discharge registers. International Classification of diseases, Ninth Edition, Clinical Modification codes was used to identify major CV outcomes (i.e., first event of myocardial infarction, coronary revascularization, stroke, carotid revascularization, ulcer, gangrene, amputation, and peripheral revascularization) and coronary artery events (i.e., first event of myocardial infarction or coronary revascularization) (Additional file 1: Table S1). All events occurred between the date of enrollment and the end of follow-up, or the date of death were considered as incident.

### Statistical analyses

Data are expressed as median (interquartile range, IQR) and/or mean ± SD for continuous variables, and number of cases and percentage for categorical variables. Continuous variables were compared by Student’s *t*-test or one-way ANOVA for normally distributed ones. Wilcoxon Sum-of-Ranks (Mann–Whitney) U test or Kruskal–Wallis tests were used for variables with skewed distribution. Pearson χ^2^ or the Fisher exact tests were applied to categories. For post-hoc comparisons, Scheffe’s test or Tamhane’s test, Mann–Whitney U test, and χ^2^ test were used for normally distributed, not-normally distributed, and categorical variables, respectively. The Spearman’s rank-order correlation was run to determine the strength and the direction of associations between two variables measured on ordinal scale.

Logistic regression analyses were used to examine the association between FLI and risk of prevalent microvascular complications after adjustment for diabetes-related variables and other potential confounding factors. Four logistic regression models were performed as follows: the first model was unadjusted; model 1 was adjusted for age and sex; model 2 was adjusted for age, sex, DD, HbA1c, smoking habits, hypertension, treatment with lipid-lowering agents and prior CV events; model 3 was like model 2 additionally adjusted for eGDR; model 4 like model 3 further adjusted for all other microvascular complications. Covariates were chosen as potential confounding factors based on their significance in univariate analyses or based on their biological plausibility.

Crude mortality rates and incidence of outcomes were described as events per 1,000 patient-years (PYs), with 95% exact Poisson Confidence Intervals (CI). Time to all-cause death or to each first outcome was plotted according to FLI categories as Kaplan–Meier (K-M) curves and comparisons were made using the log rank test. Associations between FLI categories and outcomes were tested by Cox regression analyses. The proportional hazard assumptions were checked, and none have been violated. Univariate and multivariate Cox proportional hazard models were used to identify the effect of FLI independently of key covariates, i.e., the ST1-RE (model 1), or ST1-RE and prior CV events (model 2). Models 1 and 2 were also run including the EURO-RE instead of ST1-RE. Furthermore, all models were re-run to include risk engines as continuous variables instead of categorical ones. Finally, Cox regression analyses were performed including age, sex, DD, HbA1c, smoking, LDL-cholesterol, HDL-cholesterol, ACR, eGFR, hypertension (model 1) and prior CV events (model 2) instead of ST1-RE or EURO-RE. To test the robustness of the associations between FLI (stratified as FLI < 60—ref.—and FLI ≥ 60) and outcomes, we have also calculated the Hepatic Steatosis Index (HSI = 8 × [ALT/AST ratio] + BMI [+ 2, if female; + 2, if the presence of diabetes]) [16] and added this index in the regression models that included ST1-RE and prior CV events as covariates. Results of Cox regressions are expressed as Hazard Ratio (HR) and 95% Confidence Interval (CI). All statistical analyses have been performed using SPSS package 25.0 version (IBM SPSS, Chicago, IL).

## Results

The main anthropometric and clinical characteristics of the whole study cohort and as stratified by FLI categories are reported in Table 1. Out of 774 participants, 515 subjects (66.5%) had a FLI < 30 (non-hepatic steatosis), 169 (21.8%) a FLI 30–59 (intermediate group), and 90 (11.6%) a FLI ≥ 60 (hepatic steatosis). Male gender, age, diabetes duration, total- and LDL-cholesterol, use of lipid-lowering agents, and rate of prior CV events were all higher, and eGFR levels lower in FLI 30–59 and FLI ≥ 60 compared with FLI < 30. Fasting glucose, HbA1c, and daily insulin dose were higher in FLI ≥ 60 than in FLI < 30. ACR was higher in FLI ≥ 60 than in the other FLI strata. A progressive increase through the FLI strata was apparent for systolic and diastolic BP, fibrinogen, uric acid, use of BP-lowering agents and RAS blockers and rate of hypertension, whereas a progressive decrease for HDL-cholesterol was evident. A trend for an increase across FLI strata was also observed for liver enzymes. FLI was inversely correlated (r = − 0.690, p < 0.0001) with eGDR (Additional file 1: Figure S1), with progressively lower eGDR levels (i.e., higher insulin resistance) across the three FLI categories (Table 1).

**Table 1:** Baseline clinical characteristics of the study cohort, both overall and according to FLI categories

	All patients (n = 774)	FLI < 30 (n = 515)	FLI 30–59 (n = 169)	FLI ≥ 60 (n = 90)	p
Men/women, n (%)	407/367 (52.6/47.4)	234/281 (45.4/54.6)	108/61*** (63.9/36.1)	65/25*** (72.2/27.8)	< 0.0001
Age, years	40.2 ± 11.7	37.8 ± 10.7	45.2 ± 12.1 ***	44.3 ± 12.1 ***	< 0.0001
Age at diabetes diagnosis, years	20.9 ± 10.9	20.2 ± 10.9	22.6 ± 10.6	21.8 ± 10.8	0.031
Duration of diabetes, years	19.4 ± 12.2	17.7 ± 11.6	22.7 ± 12.8 ***	22.4 ± 12.8 **	< 0.0001
BMI, kg/m^2^	24.8 ± 3.6	23.1 ± 2.3	27.0 ± 2.0	30.4 ± 3.7	
Waist circumference, cm	90.8 ± 10.7	85.5 ± 7.1	98.0 ± 6.2	107.5 ± 9.4	
Smoking habits: non-smokers, current smokers, n (%)	549/225 (70.9/29.1)	365/150 (70.9/29.1)	118/51 (69.8/30.2)	66/24 (73.3/26.7)	
Fasting glucose, mmol/l	9.44 ± 4.56	9.14 ± 4.48	9.72 ± 4.51	10.67 ± 4.91 *	0.009
HbA_1c_, %	7.83 ± 1.18	7.75 ± 1.25	7.91 ± 0.96	8.14 ± 1.09 *	< 0.0001
HbA_1c_, mmol/mol	62.1 ± 12.9	61.2 ± 13.6	62.9 ± 10.5	65.5 ± 11.9 *	< 0.0001
Systolic BP, mmHg	127 ± 18	122 ± 16	133 ± 18 ***	140 ± 16 *** ^††^	< 0.0001
Diastolic BP, mmHg	73 ± 9	72 ± 8	75 ± 8 ***	79 ± 11 *** ^††^	< 0.0001
Total cholesterol, mmol/l	4.84 ± 0.88	4.73 ± 0.84	5.06 ± 0.86 ***	5.07 ± 1.02 **	< 0.0001
LDL cholesterol, mmol/l	3.01 ± 0.76	2.88 ± 0.72	3.26 ± 0.75 ***	3.27 ± 0.84 ***	< 0.0001
HDL cholesterol, mmol/l	1.58 (1.35–1.87)	1.66 (1.40–1.94)	1.50 *** (1.27–1.85)	1.36 *** ^††^ (1.15–1.59)	< 0.0001
Triacylglycerol, mmol/l	0.85 (0.65–1.14)	0.75 (0.60–0.95)	1.07 (0.84–1.43)	1.33 (0.90–2.25)	
ALT, U/l	22.3 ± 31.8	18.9 ± 12.1	24.1 ± 16.5	38.0 ± 84.2 *** ^††^	< 0.0001
AST, U/l	20.0 ± 10.8	18.6 ± 8.3	21.9 ± 12.9 **	24.6 ± 16.0 ***	< 0.0001
Gamma-GT, U/l	20.6 ± 33.3	14.1 ± 7.1	24.9 ± 22.9	49.6 ± 84.6	
Fibrinogen, µmol/l	9.89 ± 2.00	9.57 ± 1.89	10.25 ± 1.75 **	11.00 ± 2.47 *** ^†^	< 0.0001
Albumin-to-creatinine ratio (ACR), mg/mmol	0.49 (0.26–1.00)	0.47 (0.25–0.89)	0.46 (0.26–0.97)	0.83 *** ^†††^ (0.37–3.15)	< 0.0001
eGFR, CKD-EPI, ml/min/1.73 m^2^	102.5 ± 17.4	105.0 ± 16.8	98.1 ± 15.3 ***	96.7 ± 21.2 ***	< 0.0001
Uric acid, µmol/l	223.6 ± 67.4	209.8 ± 57.1	235.9 ± 65.9 ***	279.4 ± 88.7 *** ^†††^	< 0.0001
eGDR, mg/kg/min	8.29 (5.54–9.31)	8.89 (7.37–9.65)	6.67 *** (4.37–8.19)	3.96 *** ^†††^ (3.34–6.86)	< 0.0001
Daily insulin dose, IU/kg body weight	0.66 ± 0.20	0.65 ± 0.19	0.68 ± 0.19	0.73 ± 0.27 **	0.001
Treatment with BP-lowering agents, n (%)	151 (19.5)	65 (12.6)	49 (29.0) ***	37 (41.1) *** ^†^	< 0.0001
Treatment with RAS blockers, n (%)	136 (17.6)	57 (11.1)	43 (25.4) ***	36 (40.0) *** ^†^	< 0.0001
Treatment with lipid-lowering agents, n (%)	100 (12.9)	37 (7.2)	39 (23.1) ***	24 (26.7) ***	< 0.0001
Treatment with metformin, n (%)	46 (5.9)	21 (4.1)	10 (5.9)	15 (16.7) *** ^††^	< 0.0001
Treatment with aspirin, n (%)	50 (6.5)	10 (1.9)	24 (14.2) ***	16 (17.8) ***	< 0.0001
Hypertension, n (%)	270 (34.9)	126 (24.5)	83 (49.1) ***	61 (67.8) *** ^††^	< 0.0001
Retinopathy: no retinopathy/non advanced/advanced, n (%)	452/202/120 (58.4/26.1/15.5)	334/123/58 (64.9/23.9/11.3)	80/55/34 *** (47.3/32.5/20.1)	38/24/28 *** (42.2/26.7/31.1)	< 0.0001
Distal Symmetric Polyneuropathy, n (%)	68 (8.8)	30 (5.8)	23 (13.6) **	15 (16.7) ***	< 0.0001
Diabetic kidney disease, n (%)	82 (10.6)	36 (7.0)	19 (11.2)	27 (30.0) *** ^†††^	< 0.0001
Major adverse cardiovascular events, n (%)	41 (5.3)	11 (2.1)	18 (10.7) ***	12 (13.3) ***	< 0.0001
ST1-RE: 10-year risk: < 10%/10–20%/ > 20%, n (%)	459/188/127 (59.3/24.3/16.4)	358/111/46 (69.5/21.6/8.9)	65/55/49 *** (38.5/32.5/29.0)	36/22/32 *** (40.0/24.4/35.6)	< 0.0001
EURO-RE: 10-year risk: < 10%/10–20%/ > 20%, n (%)	501/171/102 (64.7/22.1/13.2)	400/87/28(77.7/16.9/5.4)	70/55/44 *** (41.4/32.5/26.0)	31/29/30 *** (34.4/32.2/33.3)	< 0.0001

Quantitative variables are shown as mean ± SD or median (IQR)

^*^ p < 0.05; ** p < 0.01; *** p < 0.001 vs FLI < 30

^†^ p < 0.05; ^† †^p < 0.01; ^†††^ p< 0.001 vs FLI 30–59

By ST1-RE, distribution of 10-year risk strata < 10%, 10–20% and ≥ 20% was 59.3, 24.3, and 16.4%, respectively; based on the EURO-RE, the distribution was 64.7, 22.1, and 13.2%, respectively (Table 1). Concordance between the two risk engines in attribution of risk strata was 82.8%. For both risk engines, the prevalence of subjects at intermediate or high risk increased across the FLI categories (p < 0.0001).

### FLI and microvascular complications at baseline

In the entire cohort, 322 subjects (41.6%) had diabetic retinopathy (DR), 68 (8.8%) had distal symmetric polyneuropathy (DSP), and 82 (10.6%) had diabetic kidney disease (DKD) (Table 1). The prevalence of DR, and in particular advanced DR, as well as the prevalence of DSP were higher in FLI 30–59 and FLI ≥ 60 compared to FLI < 30 (Table 1). Rate of DKD was higher in FLI ≥ 60 than in the other FLI strata. By univariate logistic regression analysis (Table 2), FLI ≥ 60 was associated with an approximately sixfold increased risk of prevalent DKD (OR 5.70, 95% CI 3.24–10.02, p < 0.0001). This association remained significant after adjustment for age and sex (model 1). The strength of association was only partially attenuated upon further adjustment for DD, HbA1c, smoking habits, hypertension, treatment with lipid-lowering agents, and prior CV events (model 2). Additional adjustment for eGDR (model 3) and, in model 4 also for microvascular complications, did not affect the association between FLI and prevalent DKD. In this more comprehensive model, independent covariates of DKD were HbA1c, hypertension, DSP, and the presence of any DR (Table 2).

**Table 2:** Logistic regression analyses: association between FLI categories and the risk of prevalent microvascular complications

	Odds ratio	95% CI	p
Diabetic kidney disease			
FLI ≥ 60 category			
Unadjusted model	5.70	3.24–10.02	< 0.0001
Adjusted Model 1	4.47	2.49–8.01	< 0.0001
Adjusted Model 2	2.47	1.28–4.77	0.007
Adjusted Model 3	2.92	1.19–7.19	0.020
Adjusted Model 4	2.77	1.10–6.96	0.030
*Other independent predictors in adjusted Model 4*			
HbA1c	1.45	1.09–1.91	0.010
Hypertension	5.02	1.42–17.76	0.012
Distal symmetric polyneuropathy	2.34	1.19–4.61	0.014
Any diabetic retinopathy	2.44	1.24–4.79	0.010
Any retinopathy			
FLI ≥ 60 category			
Unadjusted model	2.52	1.60–3.98	< 0.0001
Adjusted Model 1	1.92	1.17–3.16	0.010
Adjusted Model 2	Not selected as independent covariate		
Distal symmetric polyneuropathy			
FLI ≥ 60 category			
Unadjusted model	3.23	1.66–6.93	0.001
Adjusted Model 1	Not selected as independent covariate		

Other covariates included in the multivariate logistic regression models, along with FLI categories, were as follows: Model 1: age and sex; Model 2: age, sex, diabetes duration, HbA1c, smoking habits, hypertension, treatment with lipid-lowering agents, prior cardiovascular events; Model 3: as Model 2 plus eGDR; Model 4: as Model 3 plus other microvascular complications. For diabetic kidney disease, p values associated with FLI 30–59 category were not ever significant in all models. For any retinopathy and for peripheral polyneuropathy, in unadjusted models, p values for the FLI 30–59 category were < 0.0001 and < 0.001, respectively, and not significant in adjusted Model 1

In a univariate regression analysis, FLI ≥ 60 was associated with a 2.5-fold increased risk of prevalent DR and a 3.2-fold higher risk of DSP, though these associations did not persist in the adjusted models.

Finally, when HSI > 36 was added to FLI categories (FLI stratified as < 60 vs ≥ 60) as a covariate of microvascular complications, the former was never selected as an independent predictor of diabetic kidney disease, any retinopathy or peripheral polyneuropathy, while the role of FLI was confirmed (data not shown).

### FLI, all-cause mortality and incidence of cardiovascular events

**Fig. 1:**
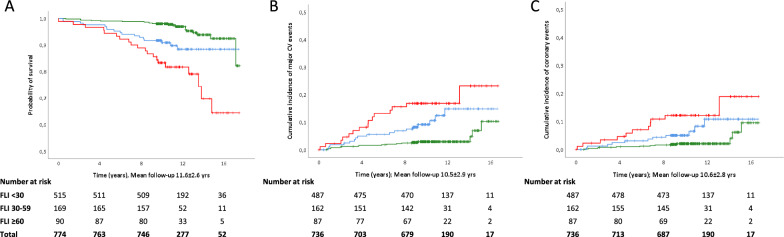
Kaplan–Meier curves for **A** all-cause death (K-M Log rank 40.367, p < 0.0001) and cumulative incidence of **B** major vascular (K-M Log rank 29.161, p < 0.0001) and **C** coronary events (K-M Log rank 19.322, p < 0.0001) by FLI categories. FLI < 30: green line, FLI 30–59: blue line, FLI ≥ 60: red line

During a mean follow-up of 11.6 ± 2.6 years, there were 57 deaths (7.4%) with an incidence density of 6.40 × 1000 PYs. Mortality rate (3.9, 10.1, and 22.2%) and incidence density (3.35, 8.67, and 19.16 × 1000 PYs) increased across the three FLI categories (p < 0.0001; Fig. 1A). In unadjusted Cox regression, with FLI < 30 as reference, risk of death increased in FLI 30–59 with an HR of 2.85 (95% CI 1.49–5.45; p = 0.002), and in FLI ≥ 60 with an HR of 6.07 (3.27–11.29; p < 0.0001).

In the attempt to establish the independent effect of FLI above and beyond existing CV risk, adjustments were performed by two independent type 1 diabetes specific risk scores as well as by including prior CV events. After adjustment for ST1-RE (model 1), HRs for death was 1.52 (0.78–2.97) for intermediate FLI and 3.04 (1.59–5.82) for FLI ≥ 60. Inclusion of prior CV events among covariates (model 2) modified HRs only slightly (Table 3). Adjustment for the EURO-RE confirms the independent role of FLI in all the models described above (Additional file 1: Table S2). Results did not change when engine scores were included as continuous variables instead of risk categories (Additional file 1: Table S3). Finally, the role of FLI was confirmed when diabetes-related variables and CV risk factors were included in the regression models (Additional file 1: Table S4).

**Table 3:** Survival analysis and incidence analysis of major CV and coronary events by Cox proportional hazards regression according to FLI categories at baseline independently of ST1-RE (model 1) or ST1-RE and prior CV events (model 2)

	*Model 1*			*Model 2*		
	HR	95% CI	p	HR	95% CI	p
*All-cause mortality*						
FLI categories			0.003			0.004
FLI < 30	1			1		
FLI 30–59	1.52	0.78–2.97	0.222	1.48	0.75–2.90	0.256
FLI ≥ 60	3.04	1.59–5.82	0.001	2.95	1.54–5.67	0.001
ST1-RE categories			< 0.0001			< 0.0001
10-year risk < 10%	1			1		
10-year risk 10–19%	3.51	1.43–8.64	0.006	3.46	1.41–8.52	0.007
10-year risk ≥ 20%	14.09	6.38–31.10	< 0.0001	13.00	5.81–29.10	< 0.0001
Prior CV events	–			1.56	0.78–3.10	0.209
*Major CV events*						
FLI categories			0.012			0.042
FLI < 30	1			1		
FLI 30–59	1.80	0.90–3.61	0.096	1.53	0.78–3.16	0.204
FLI ≥ 60	2.98	1.45–6.13	0.003	2.55	1.23–5.27	0.012
ST1-RE categories			< 0.0001			< 0.0001
10-year risk < 10%	1			1		
10-year risk 10–19%	3.50	1.44–8.52	0.006	3.19	1.31–7.77	0.011
10-year risk ≥ 20%	10.77	4.76–24.40	< 0.0001	7.36	3.15–17.20	< 0.0001
Prior CV events	–			5.51	2.88–10.55	< 0.0001
*Coronary events*						
FLI categories			0.031			0.073
FLI < 30	1			1		
FLI 30–59	1.63	0.71–3.75	0.247	1.34	0.58–3.12	0.495
FLI ≥ 60	3.07	1.33–7.08	0.009	2.62	1.12–6.10	0.026
ST1-RE categories			< 0.0001			0.004
10-year risk < 10%	1			1		
10-year risk 10–19%	3.14	1.19–8.33	0.021	2.88	1.08–7.66	0.034
10-year risk ≥ 20%	7.86	3.16–19.56	< 0.0001	5.09	1.94–13.33	0.001
Prior CV events	–			5.84	2.63–12.94	< 0.0001

Over a mean follow-up of 10.5 ± 2.9 years, 49 major CV events (6.7%) occurred in 736 individuals for whom data were retrievable, with an incidence density of 6.35 × 1000 PYs. The rate of major CV events (3.5–10.5 and 17.2%, p < 0.0001, Fig. 1B) and incidence density (3.32, 9.99, and 16.42 × 1000 PYs) increased significantly across FLI categories. In unadjusted Cox regression, the risk of major CV events increased in FLI 30–59 (HR 3.24, 95% CI 1.65–6.34; p = 0.001) and in FLI ≥ 60 (5.41, 2.70–10.83; p < 0.0001). After adjustment for ST1-RE (model 1), HRs for major CV events was 1.80 (0.90–3.61) for FLI 30–59 and 2.98 (1.45–6.13) for FLI ≥ 60. These HRs were only slightly modified after inclusion of prior CV events as covariate (model 2, Table 3). Adjustment for the EURO-RE instead of ST1-RE confirmed the independent role of FLI ≥ 60 in all models (Additional file 1: Table S2). Again, results were similar when engine scores were included as continuous variables (Additional file 1: Table S3). The association of FLI ≥ 60 with major CV events was confirmed when diabetes-related variables and CV risk factors were used in the regression models (Additional file 1: Table S4).

Over a mean follow-up of 10.6 ± 2.8 years, 35 coronary events (4.8%) occurred (incidence density of 4.50 × 1000 PYs). Rate of coronary events (2.7, 6.8, and 12.6%; p < 0.0001, Fig. 1C) and incidence density (2.51, 6.41, and 11.93 × 1000 PYs) increased across FLI categories. In unadjusted Cox regression, the risk of coronary events increased in FLI 30–59 (HR 2.74, 95% CI 1.23–6.12; p = 0.014), and in FLI ≥ 60 (5.10, 2.28–11.39; p < 0.0001). After adjustment for ST1-RE (model 1), HRs for coronary events were 1.63 (0.71–3.75) for FLI 30–59 and 3.07 (1.33–7.08) for FLI ≥ 60. Inclusion of prior CV events with all other covariates (model 2) did not affect the associated risk (Table 3). The independent role of FLI ≥ 60 was confirmed after adjustment for the EURO-RE instead of ST1-RE (Additional file 1: Table S2), or when the engine scores were included as continuous variables rather than risk categories (Additional file 1: Table S3).

In Cox regression analyses including HSI among covariates in addition to FLI (< 60 vs ≥ 60), ST1RE categories and, in Model 2, prior CV events, HSI did not enter as an independent covariate of all-cause mortality neither of major CV events, whereas FLI ≥ 60 was retained as an independent predictor of both outcomes (Additional file 1: Table S5).

## Discussion

In this > 10-year prospective study carried out in a single center cohort of people with type 1 diabetes, the Fatty Liver Index was an independent risk factor for prevalent DKD, incident CV events, and all-cause mortality. In particular, the micro- and macrovascular risk increased across categories of FLI, with FLI ≥ 60 being associated with a higher rate of complications and mortality independently of several cardio-metabolic risk factors. To test the robustness of these associations, we applied regression models including two risk scores specifically validated in longitudinal population studies for type 1 diabetes. These scores are based on the most common CV risk factors [14, 15]. Despite these thorough adjustments, a FLI score ≥ 60 remained independently associated with all outcomes. Finally, even the inclusion of prior CV events, a well-known powerful CV risk factor, did not affect the risk associated with a FLI ≥ 60.

The association between FLI and DKD at baseline is not surprising considering the growing evidence linking NAFLD and the risk of microvascular complications already shown in type 2 diabetes [17]. In the Valpolicella Heart Diabetes Study, NAFLD, diagnosed by ultrasonography, was associated to a nearly twofold increase of the risk of prevalent DKD or advanced (proliferative or laser-treated) DR [18]. A strong association between any degree of DR and NAFLD has been confirmed in the National Health and Nutrition Examination Survey-III [19]. As far as FLI is concerned, a relationship with incident chronic kidney disease has been reported in subjects with prediabetes and diabetes [20, 21].

In contrast, data in type 1 diabetes are more limited. Ultrasound-diagnosed NAFLD was reported to be associated with higher prevalence of DKD, DR or DSP [22–24]. Our findings, based on FLI stratification, agree with those observations, at least as far as DKD is concerned. The progressive increase in DKD risk across the FLI strata is in keeping with the progressive increase in adjusted ORs for DKD reported by Targher et al. [24]. At variance with those studies [22, 24], however, we found no association between FLI and DR or DSP. Therefore, our findings are more in line with those of a recent meta-analysis showing increased DSP prevalence in subjects with type 2 diabetes with NAFLD, but not in those with type 1 diabetes [25].

Previous cross-sectional studies showed an association of eGDR with DKD [26]. In our cohort, eGDR was inversely related to FLI, yet the latter was a predictor of DKD independent of eGDR. In summary, accumulating evidence indicates that NAFLD should be included among the risk factors for DKD even in type 1 diabetes [17, 27]. Consistently, at least one prospective study in type 1 diabetes demonstrated that NAFLD is strongly associated with an increased incidence of DKD independently of traditional cardio-renal risk factors [28].

Data on the burden of NAFLD on cardiovascular complications in type 1 diabetes also are limited, and the few available are conflicting. In 250 adults with type 1 diabetes, NAFLD, diagnosed using ultrasonography, was associated with an increased prevalence of CV disease (a composite of coronary, cerebrovascular, and peripheral vascular diseases), independently of conventional confounding factors [29]. These data have been confirmed in a different cohort of 343 subjects with type 1 diabetes after adjustment for the traits of the metabolic syndrome [30]. Moreover, in a retrospective cohort study of 286 subjects with type 1 diabetes followed over a mean period of 5.3 years, ultrasound-diagnosed NAFLD was associated with an approximately eightfold increased incidence of a combined endpoint including nonfatal ischemic heart disease, nonfatal ischemic stroke and coronary, carotid, or peripheral artery revascularization procedures. This association was independent of age, sex, BMI, smoking, DD, HbA1c, dyslipidemia, hypertension, CKD, prior ischemic heart disease and serum GGT levels [31].

So far, ours is the largest study evaluating the impact of NAFLD (i.e., FLI ≥ 60) on all-cause mortality (n = 774) and incident major CV outcomes (n = 736) over the longest follow-up period (mean 11.6 years for death). In keeping with Mantovani et al. [31], who used ultrasonography for NAFLD diagnosis, we found that a FLI ≥ 60 was an independent predictor of all-cause mortality as well of incidence of major CV outcomes and coronary events. This association remained an independent one even after inclusion in the regression models of the ST1-RE or EURO-RE scores [14, 15], both as risk categories and continuous covariates. Our definition of CV outcomes is almost superimposable to the one adopted by Mantovani et al. [31], therefore allowing comparison of the performance of FLI with ultrasonography diagnosis of NAFLD. With FLI ≥ 60 a HR of 5.41 for CV events was calculated, which is not so far from the HR of 8.16 reported by Mantovani et al. [31]. Therefore, a simple score as the FLI, which is based on readily available markers, may offer a simple, cheap, and effective opportunity for a more comprehensive risk prediction in people with type 1 diabetes.

Multiple mechanisms can concur in determining the negative impact of NAFLD on diabetic complications. Across the FLI strata, insulin resistance, lipid profile, blood pressure, glycemic control all worsened. Moreover, fatty liver has been shown to worsen inflammation [5, 32]. All these are well-known risk factors for DKD and CV disease. Yet, the worse overall risk profile of these subjects doesn’t seem to account for the entire negative effect of NAFLD since high FLI remained an independent predictor of complications and all-cause mortality. In summary, our results support the concept that NAFLD, as it has been found in the general population [33], in people with overt [34] and new-onset type 2 diabetes [35], is an independent risk factor for CV events and overall mortality in type 1 diabetes.

In interpreting our results, some limitations should be taken into consideration. We have used FLI as a proxy for NAFLD, the best validated score to detect steatosis [36] also endorsed by guidelines [7]. Yet, this score has been mainly used for detecting the presence rather than establishing the severity of liver steatosis. Ultrasound, or even better, MR should be used, though these approaches may have substantial limitations on large scale screenings. In subjects with type 1 diabetes, FLI ≥ 60 was found to correlate well with MR spectroscopy with an area under the receiver operating characteristic (ROC) curve of 0.86 [9]. FLI, on the other hand, though simple and not expensive, may be influenced by several factors [27]. Triglyceride levels are included in the FLI, so that transient changes in their concentrations due to worsening of glycemic control or relative hypoinsulinemia may affect the reliability of the index. In our cohort, a satisfactory cardiometabolic risk was apparent with no more than 12% of the participants with HbA1c > 9.0% and 7.5% with triglycerides > 1.69 mmol/L, with an overall poor correlation between HbA1c and triglycerides (r = 0.18). FLI doesn’t track fibrosis, and whether a fibrosis score may offer similar or better risk assessment remains to be established. Moreover, the cross-sectional assessment of the relationship between FLI and microvascular complications prevents from drawing conclusions about temporal or causal relationships between NAFLD and DKD, DR, or DSP. As far as outcomes, we registered a low number of events (57 death and 49 major CV events), making somewhat difficult a fully appropriate comprehensive multivariate analysis. In particular, some caution may be used in interpreting the association between FLI ≥ 60 and coronary events due to the relatively small number of these events (i.e., 35 patients with incident coronary events). Yet, this doesn’t undermine the strong association between FLI and all-cause mortality. Finally, a further limitation of the study is the lack of data on time-varying exposure to glucose control and cardiovascular risk factors during follow-up, as well lack of data about severe hypoglycemic events at baseline or during the follow-up observation.

Notwithstanding these limitations, our study has some strengths. Subjects have been consecutively screened and enrolled without any preselection. In contrast, imaging studies and, even more, liver biopsy studies are prone to selection bias. Such a preselection may lead to overestimation of the prevalence of NAFLD, which may account for a relative lower rate of NAFLD (namely FLI ≥ 60) in our cohort (11.6%) as compared with those based on ultrasound examination or other diagnostic modalities (19.3%, 95% CI 12.3–27.5) [8, 27]. Of note, our study assessed a relatively large population over a long follow-up period and adjusted in regression analysis models for risk engines specifically developed for type 1 diabetes. Finally, a further strength of our study relies in the observation that FLI is retained as an independent covariate of presence of microvascular complications, mainly DKD, as well of all-cause mortality and incidence of major CV events even when, to test the robustness of associations, HSI was added among confounders.

## Conclusions

In summary, our results clearly show that high FLI is consistently associated with an increased incidence of vascular events and all-cause mortality. Although further validation of NAFLD as an independent CV risk factor may be required, particularly using more direct diagnostic tools, our findings call for a need for NAFLD screening even in people with type 1 diabetes.

### Supplementary Information

**Additional file 1: Table S1.** International Classification of Diseases (ICD-9) system codes collected during follow-up. **Table S2.** Survival analysis and incidence analysis of major CV and coronary events by Cox proportional hazards regression according to FLI categories at baseline independently of EURO-RE (model 1) or EURO-RE and prior CV events (model 2). **Table S3.** Survival analysis and incidence analysis of major CV and coronary events by Cox proportional hazards regression according to FLI categories at baseline independently of ST1-RE or EURO-RE as continuous variables (model 1) or ST1-RE or EURO-RE as continuous variables and prior CV events (model 2). **Table S4.** Survival analysis and incidence analysis of major CV and coronary events by Cox proportional hazards regression according to FLI categories at baseline independently of several risk factors (model 1) or several risk factors and prior CV events (model 2). **Table S5.** Survival analysis and incidence analysis of major CV events by Cox proportional hazards regression according to HSI categories (≤36 vs >36) and FLI categories (<60 vs ≥60) at baseline independently of ST1-RE (model 1) or ST1-RE and prior CV events (model 2). **Figure S1.** Association between eGDR and Fatty Liver Index at baseline in the entire cohort.

## Data Availability

The datasets used and/or analyzed during the current study are available from the corresponding author on reasonable request. All data relevant to the study are included in this published article (and its supplementary information).

## Abbreviations

ACR: Urinary albumin to creatinine ratio
ALT: Alanine aminotransferase
AST: Aspartate aminotransferase
BMI: Body mass index
BP: Blood pressure
CHD: Coronary heart disease
CV: Cardiovascular
DD: Duration of diabetes
eGDR: Estimated glucose-disposal rate
EURO-RE: EURODIAB prospective complication study risk engine
FLI: Fatty liver index
GGT: Gamma-glutamyl transferase
HSI: Hepatic steatosis index
K-M: Kaplan–Meier
MRI: Magnetic resonance imaging
NAFL: Non-alcoholic fatty liver
NAFLD: Non-alcoholic fatty liver disease
NASH: Non-alcoholic steatohepatitis
PYs: Patient-years
ST1-RE: Steno Type 1 Risk Engine
WC: Waist circumference
