# Supplementary material for: Fatty liver index is an independent risk factor for all-cause mortality and major cardiovascular events in type 1 diabetes: an 11-year observational study
Source: Cardiovasc Diabetol. 2024 Feb 28;23:85. doi: 10.1186/s12933-024-02171-9 (PMC10902974; doi:10.1186/s12933-024-02171-9)

Fatty Liver Index Is an Independent Risk Factor for All-cause Mortality and Major Cardiovascular Events in Type 1 Diabetes: an 11-Year Observational Study

Supplementary Information: Additional file 1

Table S1 – International Classification of Diseases (ICD-9) system codes collected during follow-up. p 2

Table S2 – Survival analysis and incidence analysis of major CV and coronary events by Cox proportional hazards regression according to FLI categories at baseline independently of EURO-RE (model 1) or EURO-RE and prior CV events (model 2). p 3

Table S3 – Survival analysis and incidence analysis of major CV and coronary events by Cox proportional hazards regression according to FLI categories at baseline independently of ST1-RE or EURO-RE as continuous variables (model 1) or ST1-RE or EURO-RE as continuous variables and prior CV events (model 2). p 4

Table S4 – Survival analysis and incidence analysis of major CV and coronary events by Cox proportional hazards regression according to FLI categories at baseline independently of several risk factors (model 1) or several risk factors and prior CV events (model 2). pp 5–6

Table S5 – Survival analysis and incidence analysis of major CV events by Cox proportional hazards regression according to HSI categories (≤36 vs >36) and FLI categories (<60 vs ≥60) at baseline independently of ST1-RE (model 1) or ST1-RE and prior CV events (model 2). p 7

Figure S1 – Association between eGDR and Fatty Liver Index at baseline in the entire cohort. p 8

Table S1 – International Classification of Diseases (ICD-9) system codes collected during follow-up.

Coronary heart disease including acute myocardial infarction	ICD-9 410–414
Coronary revascularization	ICD-9 00.66, 36.0X, 36.1X
Stroke	ICD-9 431, 432.X, 433, 434, 436, 437.X
Carotid revascularization	ICD-9 00.61–00.65, 38.02, 38.12, 39.74
Ulcer, gangrene and amputation	ICD-9 440.23, 440.24, 84.1
Lower limb revascularization	ICD-9 38.18, 39.25, 39.50, 39.90
Intervention for aneurysm of the aorta	ICD-9 441.X, 38.04, 38.64, 39.71

The Inpatient Registry includes mandatory information on all principal and secondary hospital discharge diagnoses.

Table S2 – Survival analysis and incidence analysis of major CV and coronary events by Cox proportional hazards regression according to FLI categories at baseline independently of EURO-RE (model 1) or EURO-RE and prior CV events (model 2).

	Model 1			***Model 2***		
	**HR**	**95% CI**	**p**	**HR**	**95% CI**	**p**
All-cause mortality						
FLI categories			0.014			0.015
FLI <30	1			1		
FLI 30–59	1.24	0.62–2.48	0.539	1.24	0.62–2.47	0.549
FLI ≥60	2.54	1.30–4.95	0.007	2.51	1.29–4.91	0.007
EURO-RE categories			<0.0001			<0.0001
10 y risk <10%	1			1		
10 y risk 10–19%	2.83	1.23–6.55	0.015	2.79	1.21–6.46	0.016
10 y risk ≥20%	13.32	6.36–27.87	<0.0001	12.35	5.78–26.40	<0.0001
Prior CV events	---			1.38	0.69–2.78	0.361
Major CV events						
FLI categories			0.058			0.093
FLI <30	1			1		
FLI 30–59	1.49	0.73–3.03	0.268	1.38	0.68–2.79	0.377
FLI ≥60	2.44	1.17–5.09	0.017	2.25	1.08–4.69	0.031
EURO-RE categories			<0.0001			<0.0001
10 y risk <10%	1			1		
10 y risk 10–19%	4.30	1.84–10.05	0.001	3.79	1.61–8.93	0.002
10 y risk ≥20%	12.15	5.36–27.51	<0.0001	7.71	3.27–18.20	<0.0001
Prior CV events	---			5.12	2.65–9.89	<0.0001
Coronary events						
FLI categories			0.101			0.136
FLI <30	1			1		
FLI 30–59	1.28	0.55–2.98	0.560	1.11	0.48–2.59	0.805
FLI ≥60	2.44	1.05–5.67	0.038	2.21	0.95–5.15	0.067
EURO-RE categories			<0.0001			0.001
10 y risk <10%	1			1		
10 y risk 10–19%	6.27	2.35–16.72	<0.0001	5.61	2.08–15.11	0.001
10 y risk ≥20%	11.25	4.11–30.79	<0.0001	6.70	2.30–19.49	<0.0001
Prior CV events	---			5.60	2.51–12.47	<0.0001

Table S3 – Survival analysis and incidence analysis of major CV and coronary events by Cox proportional hazards regression according to FLI categories at baseline independently of ST1-RE or EURO-RE included as continuous variables (model 1) and of ST1-RE or EURO-RE included as continuous variables further adjusted for prior CV events (model 2).

	Model 1			***Model 2***		
	**HR**	**95% CI**	**p**	**HR**	**95% CI**	**p**
All-cause mortality						
FLI categories			0.001			0.001
FLI <30	1			1		
FLI 30–59	1.80	0.93–3.50	0.084	1.71	0.86–3.38	0.126
FLI ≥60	3.33	1.75–6.33	<0.0001	3.27	1.71–6.23	<0.0001
ST1-RE, 1%	1.06	1.04–1.07	<0.0001	1.05	1.04–1.07	<0.0001
Prior CV events	---			1.34	0.65–2.78	0.425
FLI categories			0.026			0.024
FLI <30	1			1		
FLI 30–59	1.58	0.80–3.12	0.192	1.46	0.72–2.96	0.296
FLI ≥60	2.57	1.29–5.12	0.007	2.55	1.29–5.06	0.007
EURO-RE, 1%	1.05	1.04–1.06	<0.0001	1.05	1.04–1.06	<0.0001
Prior CV events	---			1.59	0.77–3.27	0.212
Major CV events						
FLI categories			0.003			0.028
FLI <30	1			1		
FLI 30–59	2.34	1.19–4.62	0.014	1.81	0.89–3.65	0.099
FLI ≥60	3.27	1.59–6.71	0.001	2.69	1.30–5.59	0.008
ST1-RE, 1%	1.05	1.04–1.06	<0.0001	1.04	1.03–1.06	<0.0001
Prior CV events	---			5.58	2.88–10.80	<0.0001
FLI categories			0.017			0.075
FLI <30	1			1		
FLI 30–59	2.13	1.07–4.52	0.031	1.57	0.76–3.25	0.225
FLI ≥60	2.83	1.34–6.00	0.006	2.39	1.13–50.7	0.023
EURO-RE, 1%	1.05	1.03–1.06	<0.0001	1.04	1.03–1.05	<0.0001
Prior CV events	---			6.15	3.17–11.93	<0.0001
Coronary events						
FLI categories			0.022			0.073
FLI <30	1			1		
FLI 30–59	1.96	0.87–4.42	0.104	1.44	0.62–3.38	0.398
FLI ≥60	3.19	1.39–7.33	0.006	2.66	1.14–6.92	0.024
ST1-RE, 1%	1.05	1.03–1.07	<0.0001	1.04	1.02–1.06	<0.0001
Prior CV events	---			5.37	2.41–11.98	<0.0001
FLI categories			0.071			0.133
FLI <30	1			1		
FLI 30–59	1.78	0.78–4.07	0.171	1.26	0.52–3.04	0.615
FLI ≥60	2.75	1.15–6.57	0.022	2.34	0.98–5.61	0.056
EURO-RE, 1%	1.05	1.03–1.06	<0.0001	1.04	1.02–1.06	<0.0001
Prior CV events	---			5.98	2.69–13.30	<0.0001

Table S4 – Survival analysis and incidence analysis of major CV and coronary events by Cox proportional hazards regression according to FLI categories at baseline independently of several risk factors (model 1) or several risk factors and prior CV events (model 2).

	Model 1			***Model 2***		
	**HR**	**95% CI**	***P***	**HR**	**95% CI**	***P***
All-cause mortality						
FLI categories			0.021			0.019
FLI <30	1			1		
FLI 30–59	1.29	0.62–2.68	0.494	1.28	0.61–2.65	0.514
FLI ≥60	2.63	1.27–5.45	0.009	2.64	1.28–5.46	0.009
Age, 1 year	1.06	1.03–1.10	<0.0001	1.06	1.03–1.10	<0.0001
Active smoking	2.32	1.25–4.32	0.008	2.37	1.26–4.44	0.007
ACR, 1 mg/mmol	1.02	1.01–1.04	<0.0001	1.03	1.01–1.04	<0.0001
Prior CV events	---			1.25	0.58–2.70	0.573
Other variables included in the model, but not independently associated	Sex, diabetes duration, HbA1c, LDL-cholesterol, HDL-cholesterol, eGFR CKD-EPI, hypertension			Sex, diabetes duration, HbA1c, LDL-cholesterol, HDL-cholesterol, eGFR CKD-EPI, hypertension, prior CV events		
Major CV events						
FLI categories			0.030			0.051
FLI <30	1			1		
FLI 30–59	1.92	0.92–3.98	0.082	1.79	0.86–3.76	0.121
FLI ≥60	2.98	1.32–6.73	0.009	2.70	1.21–6.00	0.015
Age, 1 year	1.05	1.02–1.09	0.003	1.04	1.01–1.08	0.015
LDL-cholesterol, mmol/l	0.65	0.43–0.96	0.032			ns
Hypertension	2.87	1.35–6.09	0.006	2.55	1.17–5.54	0.019
ACR, 1 mg/mmol	1.02	1.01–1.03	0.006	1.02	1.01–1.04	0.001
Prior CV events	---			4.51	2.19–9.29	<0.0001
Other variables included in the model, but not independently associated	Sex, active smoking, diabetes duration, HbA1c, HDL-cholesterol, eGFR CKD-EPI			Sex, active smoking, diabetes duration, HbA1c, HDL-cholesterol, LDL-cholesterol, eGFR CKD-EPI		
Coronary events						
FLI categories			0.215			0.264
FLI <30	1			1		
FLI 30–59	1.37	0.57–3.32	0.484	1.18	0.48–2.89	0.723
FLI ≥60	2.30	0.90–5.91	0.084	2.08	0.82–5.28	0.125

Age, 1 year	1.05	1.01–1.09	0.009	1.04	1.01–1.09	0.027
ACR, 1 mg/mmol	1.02	1.01–1.04	<0.0001	1.03	1.01–1.04	<0.0001
Prior CV events	---			5.28	2.15–12.98	<0.0001
Other variables included in the model, but not independently associated	Sex, active smoking, diabetes duration, HbA1c, HDL-cholesterol, LDL-cholesterol, eGFR CKD-EPI, hypertension			Sex, active smoking, diabetes duration, HbA1c, HDL-cholesterol, LDL-cholesterol, eGFR CKD-EPI, hypertension		

Table S5 – Survival analysis and incidence analysis of major CV events by Cox proportional hazards regression according to HSI categories (≤36 vs >36) and FLI categories (<60 vs ≥60) at baseline independently of ST1-RE (model 1) or ST1-RE and prior CV events (model 2).

	Model 1			***Model 2***		
All-cause mortality						
FLI categories						
FLI <60	1			1		
FLI ≥60	2.52	1.44-4.39	0.001	2.52	1.44-4.39	0.001
ST1-RE categories			<0.0001			<0.0001
10-year risk <10%	1			1		
10-year risk 10–19%	3.72	1.52-9.11	0.004	3.72	1.52-9.11	0.004
10-year risk ≥20%	15.60	7.18-30.88	<0.0001	15.60	7.18-30.88	<0.0001
Prior CV events	----			--	--	--
Other variables included but not selected as independent covariate: HSI in both models, prior CV events in Model 2						
Major CV events						
FLI categories						
FLI <60	1			1		
FLI ≥60	2.26	1.21-4.20	0.010	2.04	1.09-3.80	0.026
ST1-RE categories			<0.0001			<0.0001
10-year risk <10%	1			1		
10-year risk 10–19%	3.83	1.59-9.24	0.003	3.40	1.40-8.24	0.007
10-year risk ≥20%	12.61	5.68-27.99	<0.0001	8.12	3.51-18.78	<0.0001
Prior CV events	----			5.79	3.02-11.10	<0.0001
Other variables included in both models but not selected as independent covariate: HSI						

Figure S1 – Inverse association between eGDR (estimated glucose disposal rate) and Fatty Liver Index at baseline in the entire cohort of individuals with type 1 diabetes (n=774). Spearman correlation: r=-0.690, p<0.0001.

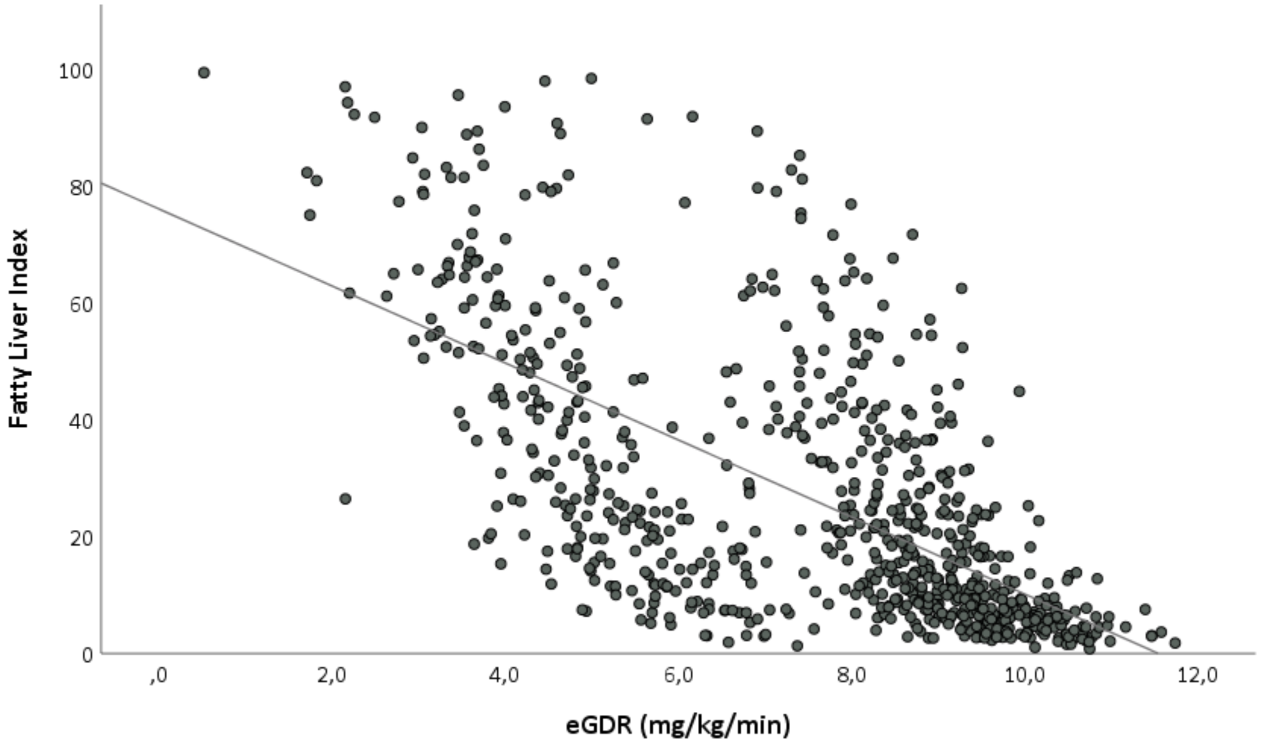

Supplement: Supplementary file 1 — Additional file 1: Table S1. International Classification of Diseases (ICD-9) system codes collected during follow-up. Table S2. Survival analysis and incidence analysis of major CV and coronary events by Cox proportional hazards regression according to FLI categories at baseline independently of EURO-RE (model 1) or EURO-RE and prior CV events (model 2). Table S3. Survival analysis and incidence analysis of major CV and coronary events by Cox proportional hazards regression according to FLI categories at baseline independently of ST1-RE or EURO-RE as continuous variables (model 1) or ST1-RE or EURO-RE as continuous variables and prior CV events (model 2). Table S4. Survival analysis and incidence analysis of major CV and coronary events by Cox proportional hazards regression according to FLI categories at baseline independently of several risk factors (model 1) or several risk factors and prior CV events (model 2). Table S5. Survival analysis and incidence analysis of major CV events by Cox proportional hazards regression according to HSI categories (≤36 vs >36) and FLI categories (<60 vs ≥60) at baseline independently of ST1-RE (model 1) or ST1-RE and prior CV events (model 2). Figure S1. Association between eGDR and Fatty Liver Index at baseline in the entire cohort. [file 12933_2024_2171_MOESM1_ESM.pdf]
